# Epidemiology, Predisposing Factors, Biomarkers, and Prevention Mechanism of Obesity: A Systematic Review

**DOI:** 10.1155/2020/6134362

**Published:** 2020-05-31

**Authors:** Melese Linger Endalifer, Gedefaw Diress

**Affiliations:** College of Health Sciences, Woldia University, Woldia, Ethiopia

## Abstract

*Background*. Globally, obesity is becoming a public health problem in the general population. Various determinants were reported by different scholars even though there are inconsistencies. Different biomarkers of obesity were identified for the prediction of obesity. Even though researchers speculate the factors, biomarkers, consequences, and prevention mechanisms, there is a lack of aggregate and purified data in the area of obesity. *Summary*. In this review, the epidemiology, predisposing factors, biomarkers, consequences, and prevention approaches of obesity were reviewed. *Key Messages*. The epidemiology of obesity increased in low-, middle-, and high-income countries. Even if the factors vary across regions and socioeconomic levels, sociodemographic, behavioral, and genetic factors were prominent for the development of obesity. There are a lot of biomarkers for obesity, of which microRNA, adipocytes, oxidative stress, blood cell profile, nutrients, and microbiota were promising biomarkers for determination of occurrence of obesity. Since the consequences of obesity are vast and interrelated, multidimensional prevention strategy is mandatory in all nations.

## 1. Introduction

The 2017 global nutrition report showed that 2 billion adults are overweight/obese and 41 million children are overweight worldwide [1]. In the last three decades, obesity increased globally; unexpectedly, it is also rising in low- and middle-income countries due to uncontrolled urbanization and nutrition transition (shifting dietary habit from traditional to westernized diet) [2, 3]. The global prevalence of overweight in children aged less than five years was increased modestly. The trend of overweight was heterogeneous in low- and middle-income countries. Meanwhile, the prevalence of obesity in children aged 2–4 years has increased moderately. In 1975, children with obesity aged 5–19 years were relatively rare, but it becomes highly prevalent in 2016 [4].

In the majority of European countries, the prevalence was increased from 10% to 40% in the last 10 years, and specifically in England, it increased more than threefolds [5].

The prevalence of obesity among reproductive-age women was 5.1% in India [6], 15.7% in Palestinian schoolchildren, and 34.8% among adult populations of Saudi Arabia [7]. The prevalence of overweight and obesity was 40.9% in Kuwait among children aged 6–8 years. Amazingly, their mothers' perceive that they had healthy weight; in contrary, those children who have normal weight were also criticized by their mothers to be unhealthy [8].

A systematic review conducted in Africa among primary school educators revealed that the continental figure of obesity increased. In this review, the magnitude of obesity was measured based on three international standards, i.e., World Health Organization (WHO), Center for Disease Control (CDC), and International Obesity Taskforce (IOTF) cutoff points. Based on the criteria mentioned above, the prevalence of obesity was 6.1% (WHO criteria), 4.0% (IOTF criteria), and 6.9% (CDC criteria) [9]. Generally, the prevalence of obesity in Africa among schoolchildren lies between 4.4% and 21.2 percent [9–11].

Another critical issue is currently the emerging sarcopenic obesity. Sarcopenic obesity is defined as loss of skeletal muscle and excess body fat accumulation. Clinically, it can be diagnosed through muscle biopsy, computed tomography or magnetic resonance spectroscopy, bioelectrical impedance analysis (BIA), and dual energy X-ray. Primarily, the consequence of sacopenic obesity end is liver cell damage either carcinogen or any abnormality [12]. It is highly prevalent in elder population even though it did not get emphasis in the majority of countries.

## 2. Predisposing Factors of Obesity

Different scholars mention a lot of predisposing factors which vary depending on geography, social conditions, political and economic factors, and human genetics. In aggregate, the commonest factors were sociodemographic, behavioral, genetic, and living in obesogenic environment.

### 2.1. Sociodemographic Factors

Based on United Nations Children's Fund (UNICEF) causes of malnutrition analysis, three causes were identified. According to the framework, the basic causes including poverty, social condition, and political, economic, ecological, and other factors were the root reason for any form of malnutrition [13]. Different literature studies explicitly identified sociodemographic factors that were highly correlated with obesity, for example, older age [2, 6], married (marital status) [14], low wealth index [6, 10, 15–17], urban residency [6, 10, 16, 18], being female [2, 9], learning in private schools [2, 9, 19, 20], easy accessibility of junk and fired or energy-dense foods and packed animal source foods due to free trade policy [2], rural to urban migration, replacement of local agribusiness with food retail [21], higher education level [6, 7, 22], and being pregnant [6, 18]. In contrary to the previous findings, a study conducted among French women shows that having a higher income, a higher occupational class, and a higher educational level and having hot water at home reduce the occurrence of obesity [23] although the pathophysiology of hot water at home and obesity occurrence was not yet studied.

Another cause of obesity mostly in developing countries is that early-life undernutrition leads to later-life obesity and metabolic disorders. The correlation between childhood under nutrition and the development of obesity in later life is idiopathic; but there is a different hypothesis stated by the scholars. Of those, the first is when there is an improvement in the socioeconomic level, and living standards and exposure to obesogenic environments outside the uterus leads to obesity. This might be imbalance between intrauterine and later life nutrient requirements. Secondly, the positive response of under nutrition in the womb to protect vital organs and the exposure to obesogenic environment may leads to obesity. Additionally, the positive response of undernutrition in the womb to protect vital organs and the exposure to obesogenic environment may lead to obesity [2].

### 2.2. Behavioral Factors (Feeding Habit and Life Style)

Nutritionists always use the following proverb to explain the effect of diet in our health “what you eat today; they determine your life tomorrow.” Dietary habit is a major determinant factor for our health, not merely to obesity.

Scientifically, consuming energy-dense food, like confectionaries, sugars, soft drinks, fats, and alcohol, were highly correlated with obesity and chronic diseases [11, 24, 25]. Different scholars mentioned that feeding habit culture [26], consuming pastry foods [26], consuming ultraprocessed food (refined carbohydrate) [21], excess alcohol consumption [10, 14], and monotonous diet or poor diet quality [11, 24, 27, 28] increase the occurrence of obesity. Eating breakfast and fruit reduces the occurrence of obesity [27], and in other words, evening snack induces obesity [29]. Furthermore, food store environment and school food environment [15] for school age children expose to obesity.

Many literature studies extensively identified that either irregular physical exercise or physical inactiveness [7, 10, 14, 15, 26, 30], watching television or prolonged screen time [27, 30], short sleep duration or shift work [15, 24, 30], stress, obesogenic environment (urbanization and industrialization) [31], smoking [10], and frequent use of a taxi for transportation [10, 32] were determinant factors for overweight/obesity.

Watching electronic screens for more than 2 hours increases the development of obesity because during simple observation, the brain does not utilize glucose and as a result, the metabolism of carbohydrate to glycogen and fat increased consistently [27, 30]. The correlation between stress and the development of obesity has different scientific perspectives. Most scholars conclude that hormonal variation may be a cause. During stress, the cortisol levels rise which is a cause for excess production of abdominal fat by increasing appetite (daily intake) [33–35].

#### 2.2.1. Genetic Factors

Evidence revealed that a family history of obesity and different genetically arranged genes were a risk for obesity [15, 26]. Genome-wide association studies (GWAS) identified that more than 250 genes/loci were associated with obesity. Of these genes, the fat mass- and obesity-associated gene (FTO) showed an important role for development of the obesity and type 2 diabetes. A study conducted among adults explicitly recognizes the correlation between these genes and a higher body mass index (BMI), fat mass index (FMI), and leptin concentrations [32, 36–38]. Almost all studies included in this review use cross-sectional study design, and majority of those studies assess obesity with the WHO standard (Table 1).

## 3. Assessment Methods of Obesity

In nutritional science, there are four basic nutritional assessment methods, i.e., anthropometric, biochemical, clinical, and dietary methods [42]. Similarly, we can also assess obesity through these methods. In this review, we discuss two nutritional assessment methods (anthropometric and biochemical) in detail.

### 3.1. Anthropometric Assessment

Obesity can be assessed through BMI, waist circumference (WC), body fat percentage (BFP), and skin fold thickness (SFT). As evidenced in the literature, the anthropometric method shows a correlation between the factors [18, 20, 43]. Recently, the classification of obesity was reevaluated and validated again with the consideration of morbidity and mortality at population level. Obesity is further classified into four categories: (1) normal weight obese (NWO), (2) metabolically obese normal weight (MONW), (3) metabolically healthy obese (MHO), and (4) metabolically unhealthy obese (MUO) [44]. The current approach is more reliable than the previous approach to predict obesity and its correlated disorders since using only BMI gives gross data which are difficult to interpret (Table 2).

#### 3.1.1. Biochemical Methods

Among the nutrition assessment methods, the biochemical method is objective and more reliable. There are two types of biochemical methods, functional and static methods. The functional method is used when there is a deficiency or an excess of nutrient which leads to functional impairment [42]. Unexpectedly, obese children have significantly lower ability to identify taste types and qualities correctly due to lesser number of fungiform papillae in the tongue [50].

## 4. Biomarkers

Biomarkers are biological indicators for certain disorders in our body. Different categories of biomarkers were reported globally. Among those, the commonly and widely implemented ones are microRNAs, inflammatory biomarkers, adipocytokines, oxidative stress, gut microbiotas, level of nutrients, and blood cell profiles.

### 4.1. Micro-RNAs

A study conducted among children identified four miRNAs overexpressed in patients with obesity (miR-222, miR-142–3, miR-140-5p, and miR-143) and two miRNAs (miR-122 and miR-34a) overexpressed in children with obesity and nonalcoholic fatty liver disease (NAFLD) and/or insulin resistance. Circulating miRNAs are promising diagnostic biomarkers of obesity and other disorders such as cardiovascular diseases [51]. Another study also reports eight miRNAs which are found in obese population, that is, PTEN gene (hsa-miR-130b-3p, hsa-miR-142-5p, hsa-miR-148a-3p, hsa-miR-21-5p, hsa-miR23a-3p, hsa-miR-26b-5p, hsa-miR-320a, and hsa-miR- 486-5p) [46, 47, 52]. The early detection of changes in circulating miRNA levels represents a promising strategy for characterizing obesity and to adjust diet. Moreover, the presence of biomarkers at the early stage is usually associated with metabolic disease or syndrome. As a result, identification of these miRNA is a good strategy for the diagnostic approach as well as to prevent the occurrences [36].

### 4.2. Inflammatory Biomarkers

Researchers depict that inflammatory biomarkers (C-reactive protein, interleukin-6, and tumor necrosis factor) were identified in obese population [46, 47].

### 4.3. Adipocytokines

Adipocytokines can play a role in the observed link between obesity and its associated morbidities. It has high predictive potential for identification of adverse cardiovascular conditions. Similarly, Plasminogen Activator Inhibitor-1 (PAI-1) was found as an independent risk factor for obesity-related metabolic disorders, even though it needs further investigation on mechanisms of action [53]. Other reviews also suggest that adiponectin, omentin, apelin, leptin, resistin, and fatty-acid-binding protein-4 were promising biomarkers for obesity [46, 47].

### 4.4. Oxidative Stress

High oxidative stress in the body is usually associated with high anthropometric measurement results, specifically BMI and waist-hip ratio [47, 53]. Among various antioxidants, F2 isoprostanes are widely used to assess lipid oxidation and have demonstrated their ability to predict biological changes and cardiovascular diseases resulting from obesity even though the specificity and sensitivity are not determined. Also, glutathione peroxidase has strong antioxidant and antiatherosclerotic properties. Additionally, complement factor 3 and Monocyte Chemoattractant Protein-1 (MCP-1) promote fat deposition and are also associated with atherosclerosis, excess fat accumulation, and adverse cardiovascular risk [53].

### 4.5. Gut Microbiota

*Helicobacter pylori* is an indicator for development of obesity; even though the exact pathophysiology is unknown, most studies correlate with gastrointestinal hormones such as leptin and ghrelin. In normal physiology, ghrelin facilitates food intake and leptin is involved in reduction of food consumption. It is evidenced that there is low serum leptin and ghrelin levels in *H. pylori*-positive patients. As a result of the level of leptin, food intake will be reduced. So its reduction may be involved in excessive dietary intake and obesity. In contrary, reduction of plasma ghrelin concentration ends with a physiological adaptation to the positive energy balance associated with obesity [47, 54].

### 4.6. Blood Cell Profile

The morbid obesity group had significantly higher platelet counts, plateletcrit (PCT) values, and platelet-to-lymphocyte ratio (PLR) values. The values of white blood cell count and red cell distribution width (RDW) were higher and statistically significant in the obese population [55].

### 4.7. Nutrients

High availability of branched-chain amino acids (BCAA), nonesterified fatty acids, organic acids, acylcarnitines, deficient 25(OH) vitamin D serum concentrations, and phospholipids was identified as potential biomarkers for obesity [56, 57].

## 5. Health Impact of Obesity

High BMI and accumulation of body fat mass are an important predictor for metabolic disorders [43]. Obesity during pregnancy leads to adverse neonatal outcomes (skeletal muscle injury, respiratory distress syndrome, injury to peripheral nervous system, bacterial sepsis, convulsion, hypoglycemia). Additionally, it increases the rate of cesarean section [58–60] and morbidity for the women [61].

Obesity is also associated with a range of comorbidities, including diabetes mellitus [7], dyslipidemia [7], hypertension [7], cardiovascular disease, obstructive sleep apnea, chronic obstructive pulmonary diseases [62], cancer [63, 64], chronic disease morbidity and mortality, premature death [24, 62], and atrial fibrillation [65]. Hypertension was also strongly associated WC, BMI, and waist hip ratio (WHR) [66].

A review conducted in the USA population shows that the magnitude of obesity among coronary heart disease patients was increased [31]. The effect of obesity varies in different age groups; a systematic review identified that students with obesity in tertiary education have low academic performance and poor achievements either due to weight gain bias stigma or metabolic disorder [67]. A 25-year longitudinal study from 1986 to 2011 conducted in America showed that baseline obesity better predicts long-term risk of cerebrovascular death in black individuals as compared to white people. More research should explore factors that explain why racial differences exist in the effects of obesity on cerebrovascular outcome. Findings also have implications for personalized medicine [4].

As indicated from another systematic review, obesity may influence the course of renal cell carcinoma (RCC) patients, although the interplay between obesity and RCC warrants a large prospective confirmation [68].

Central obesity is highly correlated with kidney injury [62, 69]. It also has significant correlation with urinary incontinency. Specifically, central obesity correlates with intra‐abdominal pressure, which exerts forces in the pelvic floor. Polycystic ovary syndrome (POS) was highly correlated with obesity which is highlighted in different clinical and epidemiological studies [70].

Obesity also leads to anatomical deformity; a study conducted in Egypt among schoolchildren explicitly shows that the occurrence of flat foot was high among obese children. The presence of flat foot leads to foot pain which is significantly manifested with increased level of adipocyte cytokines, as well as adiponectin, leptin, resistin, Il-6, and TNF-*α*, compared to subjects with normal BMI [71]. Physical inactiveness like slow waking/decreased velocities [72, 73] and mental comorbidities [72] were also other consequences of obesity.

Amazingly, obesity has significant effect on the reproduction of human. Sexual dysfunction is highly prevalent in men with severe with erectile dysfunction in diabetic patients was likely a significant contributing factor for sexual dysfunction in obese population [74–76].

Obesity leads to development of different cancers; this is why, cancer epidemiology abruptly increased worldwide. Obesity also promotes breast cancer formation [77] and formation of Barrett's esophagus (BE) [78]. BE has been defined as a pathological state in which the stratified squamous epithelium of the distal esophagus has been replaced by the metaplastic columnar epithelium with goblet cells. The formation of BE predisposes patients to esophageal adenocarcinoma (EAC) [78].

The impact of obesity varies based on the trait and biological differences like sex. Also, it has an etiological role in the death of most people globally [62]. In general, the public health significance of obesity is highly integrated with country's economic, social, and political affairs.

## 6. Prevention and Treatment Mechanism

Obesity is a disorder which occurs due to individual behaviors and the living environment. As a result of this, to prevent obesity, both legal and voluntary counseling services are mandatory. Obesity can be prevented or treated based on the following approach.

### 6.1. Nutrition Education

Nutrition education is one of the common legal approaches practiced at schools to reduce obesity in the USA. Disseminating health education and developing dietary consumption standards at organization level also have significant impact [79]; weight loss programs [70] and diabetes prevention approaches were effective programs to reduce obesity which is reported elsewhere [80]. Interventional studies which are entitled as “Healthy Primary School of the Future” implemented in Dutch are primarily focused on lunch health education, healthy diet approach, and physical activity session, lowering children's BMI z-scores [82]. Another program conducted in Brooklyn entitled as “Live Light Live Right program” is a life style intervention that uses medical assessment, nutritional education, access to physical fitness classes, and behavioral modification to reduce BMI Z-Scores [83]. Even moderate physical activity is effective to control overweight/obesity among pregnant women [83, 84].

### 6.2. Developing Nonsedentary Life Style Plan

Physical activity, reducing sedentary time, reducing fast food consumption, sleeping 7–9 hours per day, avoiding smoking, and moderate alcohol drinking habit were effective interventions to reduce obesity [85].

### 6.3. Healthy Food Subsidization and Taxation of Junk Food

The good news regarding obesity is that the government can reduce obesity by subsidization of healthy foods or increasing taxation of junk foods. This is strongly implemented in the UK; this scenario shows that an increment of price of high sugar snacks by 20% shows significant reduction in energy intake, BMI, and prevalence of obesity. As a result, increasing taxation or price for unhealthy foods is an effective approach to control obesity and their metabolic disorder [86].

### 6.4. Surgery

Metabolic and bariatric surgery in the pediatric population provides evidence-based effective treatment of severe obesity and related comorbid diseases in the USA [87].

## 7. Conclusion and Future Perspectives

Obesity is becoming a severe public health problem; its epidemiology is increasing rapidly. The exposure factors vary across different geopolitics. Primarily, living in obesogenic environments such as sedentary life style, urbanization, and rural to urban migration, consuming energy-dense foods, and physical inactivity were determinants. There are a lot of biomarkers, of which microRNAs, adipocytes, oxidative stress, and microbiota were promising for determination of obesity. Since the consequences of obesity are vast and interrelated, a multilevel prevention strategy is mandatory. For future researchers, the sensitivity of the anthropometric measurement tool was not studied. So it is better to study sensitivity and its correlation with the best promising biomarkers since they reduce health cost and facilitate early identification of obesity.

## Figures and Tables

**Table 1:** Descriptive characteristics of studies included in this review.

Authors, country	Study population (sample size)	Study design	Anthropometric used	Criteria	Prevalence of obesity	Risk factors
Al-Lahham et al. [18], Palestine	Schoolchildren (*N* = 1320)	Cross-sectional	BMI percentiles	CDC	15.7%	Urban residence and high waist circumferences
Golshevsky et al. [30], Australia	Children (*N* = 343)	Cross-sectional	BMI percentiles	CDC	No prevalence	Watching television, obstructive sleep and sleep apnea
Gokosmanoglu et al. [26], Turkey	Adolescent (*N* = 750)	Cross-sectional	BMI	WHO	4%	Irregular physical exercise, family history of obesity and consuming pastry foods
Chomba et al. [39], Tanzania	Schoolchildren (*N* = 451)	Cross-sectional	BMI percentile	WHO	12.6%	Being a girl, random sleeping time and random eating habit
Baratin et al. [40], Ghanaians	Adults (*N* = 5898)	Cross-sectional	BMI	WHO	No prevalence	Negative life events and stress at work place
Baalwa et al. [10], Uganda	Adults (*N* = 683)	Cross-sectional	BMI	WHO	2.3%	Urban residence, alcohol consumption, smoking, physical inactiveness, using vehicle for transport and richness
Addo et al. [14], Ghana	Adults (*N* = 180)	Cross-sectional	BMI	WHO	17.8%	Being physically inactive, consumption of alcohol, being married, female, older age
Karki et al. [41], Nepal	Schoolchildren (*N* = 575)	Cross sectional	BMI for age-sex	WHO	7.1%	Children mothers' high education level, having professional mother, consuming energy-dense food, having sedentary behaviors
Ganle et al. [11], Ghana	School children (*N* = 285)	Cross-sectional	BMI	WHO	21.2%	Being aged 11–16, family high education level and consumption of fizzy drinks
Firouzbakht et al. [17], Iran	Female (*N* = 680)	Cross-sectional	BMI	WHO	51.2%	Weak structural social capital
Adom et al. [9], Africa	Children (*N* = 89468)	Systematic review and meta-analysis		WHO/CDC/IOTF	6.1%, 6.9%, 4%	Urban residence and learning in private school
Al Kibria et al. [6], India	Women (*N* = 647, 168)	Cross-sectional	BMI	WHO	5.1%	Older age, ever-pregnant, ever married, being muslims, high education level, wealthy and urban residence
Al-Raddadi et al. [7], Saudi Arabia	Adult (*N* = 1419)	Cross-sectional	BMI	WHO	34.8%	No factor identified
Narciso et al. [15]	Adolescent (*N* = )	Systematic review				Genetic factors and socioeconomic factors
Sagbo et al. [27], Togo	Adolescent (*N* = 634)	Cross-sectional	BMI	IOTF	1.9%	Watching television, medium dietary diversity score
Hu et al. [20], China	Adult (*N* = 15364)	Cross-sectional	BMI	WHO	7.9%	Urban residence

**Table 2:** Phenotype of obesity and its biomarkers.

Type of obesity	Benchmarks	Biomarkers	References
Normal weight obese (NWO)	(A) BMI = 18.5–25 (normal weight) (B) Higher fasting glucose level (C) Did not have metabolic syndrome (D) High body fat percentage ✓ Men—23.5% ✓ Women—29.2%	Proinflammatory cytokines	[44–47]

Metabolically obese normal weight (MONW)	(A) BMI = 18.5–25 (normal weight) (B) High body fat percentage >30 (C) Low insulin sensitivity/hyperinsulinemia (D) Metabolic syndrome happen	Presence of steatosis, concentrations of high-density cholesterol, triglycerides, and inflammation biomarkers	[44–46, 48]

Metabolically healthy obese (MHO)	(A) BMI >30 (obese) (B) High body fat percentage >30 (C) Proper sensitivity of insulin	Elevated high sensitivity C-reactive protein (hs-CRP) and TG	[44, 45, 48, 49]

Metabolically unhealthy obese (MUO)	(A) BMI >30 (obese) (B) High body fat percentage >30 (C) Insulin resistant, diabetes mellitus	Higher TG, FBG, TG/HDL-C levels, and lower levels of HDL	[44, 45, 49]

FBG: fasting glucose; TG: triglyceride; HDL: high-density lipoprotein.
